# Editorials

**Published:** 1891-01

**Authors:** 


### HOMŒOPATHIC PHYSICIAN,

A MONTHLY JOURNAL OF

HOMŒOPATHIC MATERIA MEDICA AND CLINICAL MEDICINE.

"If our school ever gives up the strict inductive method of Hahnemann, we are lost, and deserve only to be mentioned as a caricature in the history of medicine."—CONSTANTINE HERING.

Vol. XI. JANUARY, 1891. No. 1.

### EDITORIALS.

SALUTATORY.—CONSTANTINE HERING, whom we may justly call the American Hahnemann, wrote, just before his death, those warning words which appear at the top of our page. This admonition we shall keep displayed at our mast-head, to serve as a beacon-light of warning to the foolhardy practitioner who would desert our law, the true and unerring compass of therapeutics, and trust to chance knowledge of the rocky coast or hidden sand-bars which the practitioner continually meets in his stormy crusade against disease and death.

Soon after the demise of our late teacher and guide, some friends of the departed hero and earnest workers for pure Homœopathy met to consider how his last injunction could be best obeyed, and that the life-work of this prince of workers should not be rendered nugatory nor our noble school live "as a caricature in the history of medicine."

To assist in this noble work a pure, able homœopathic journal was considered necessary. And, that this journal might exert a powerful influence for good in our school, it was determined to ask the active co-operation of the best and ablest men in our ranks. This was done; and the hearty, willing offers of assistance which came back to us were very gratifying, and, moreover, assured us of success; it was even more pleasing than this, as it proved our best men were alive to the danger and eager to meet it.

As to our course and work, we may say THE HOMŒOPATHIC PHYSICIAN—so called because the homœopathic physician represents the full complement of scientific medicine, and because Hahnemann considered it a title of the highest honor—will strive to show that the conscientious practitioner preserves intact "the strict inductive method of Hahnemann;" also, that the following are the true and essential features of Homœopathy:

The Law of the Similars,
The Single Remedy,
The Minimum Dose, the first being the unfailing law, the last two its logical corollaries.

To establish these principles, THE HOMŒOPATHIC PHYSICIAN will offer logical argument and clinical proof; all "fatal errors," made by those attempting to pervert these principles, all deviations from the strict application of *the Law*, will be courteously yet *fearlessly* combated. In short, this new advocate for professional favor will defend unflinchingly in its pages that law which has never failed its editors in the sick-room.

A large portion of its work will be clinical matter furnished by our able corps of distinguished contributors; the materia medica will be fully compared, corrected, and illustrated by the best therapeutists in the homœopathic school; current medical literature will be thoroughly scanned for interesting or instructive matter; books will be impartially reviewed; the papers will aim to be short, clear, and to the point.

The law of similars is to Homœopathy what the "vital spark" is to the human frame; crush it out and we are but a dead mass, certain to become corrupt and to decay. To preserve this law, this vital spark, should be the earnest desire of all true men and all earnest physicians. No true men could oppose such a noble work—noble, for it seeks to preserve and to perfect a science whose sole object is to relieve and cure human misery. To this work is this journal dedicated and for this purpose is it established. We ask the aid of all true homœopaths, promising to be "independent in everything, neutral in nothing."

---

The foregoing editorial written by the former editor of this journal, Dr. Edmund J. Lee, appeared exactly ten years ago in the first number of THE HOMŒOPATHIC PHYSICIAN ever issued.

The professions and promises there made have been faithfully kept, as most of our subscribers, who have been steady readers of the journal from the beginning, clearly appreciate. Thus the past career of THE HOMŒOPATHIC PHYSICIAN is in marked contrast to all the journals published as professed adherents of our school. It has never swerved from the strict line of the "inductive method of Hahnemann;" it has been "independent in everything, neutral in nothing;" it has set a shining example that other journals are now imitating. As the aim of the journal has been to teach the only successful and harmless method of healing the sick, the fact that its example *is* imitated by other journals gives us the highest satisfaction. It is a proof of the justice of our cause, the influence of our words; it is the highest compliment that can be paid us.

How many physicians have been educated in the true art by its teachings! It has been a college in itself in the instruction it has afforded to those, who, not knowing, yet desired enlightenment.

The condition of the homœopathic school at the beginning of the last decade was one of transition, a passing from the strict homœopathic practice of Hering and his contemporaneous workers into the avowedly eclectic practice of to-day. To-day we find the literature, the colleges, and the medical societies all openly professing and teaching the plainest eclecticism. Ten years ago a mongrel pretended to be a homœopathist, to-day he boastfully proclaims himself "a scientific physician," which is merely an "alias" for eclecticism. The literature of our school, nominally, homœopathic, has been steadily deteriorating, until now nothing of the true homœopathic philosophy is taught. Is there not, then, even a greater need for a strong and fearless journal to uphold the "true inductive method" of the immortal Hahnemann?

In short, every word of that editorial is even more applicable now and for the coming decade than it was ten years ago. It is the summary of our opinion of the needs of the present, an earnest promise of our intentions for the future.

W. M. J.

---

SALUTATORY.—While not competent to say just what all physicians need in a journal, tastes are so varying, we feel we are justified in saying that every one needs and should demand practical facts. Such facts as shall be of use every day, and for all time. This has been our effort in the past, and we shall continue in this course. Theories should be as playthings to the practical physician; they should have no place in the mind of a conscientious practitioner at the bedside. When one meets the sufferings of the sick there is demanded that which will give help in the quickest, safest, and most pleasant manner. Every physician should be Gradgrindian in the pursuit of practical, valuable facts, and he who possesses such should always be willing to make them known.

Notwithstanding the advantages we, as Hahnemannians, have over those who know nothing of law in the treatment of disease, efforts are being continually made to belittle the work of Hahnemann and his followers by attempts to engraft upon Homœopathy silly theories that have no connection with the law, and thus many are led away from facts, and flounder helplessly in the mire of empiricism. This is done not only by our opponents, but by many who profess to be true disciples of the master. Thus much harm is being done, and it is incumbent upon all the faithful to combat these efforts.

We know—actual proof is ours—that we possess the only *law* of cure. We know that the latest developments which are found to be true, in respect to the cause and nature of diseases, are not at variance with this law and its corollaries, and that we need only still adhere to our law to be successful in the treatment of any and all affections, from the simplest to the most malignant—even if they be due to microbes.

The microbe craze now reigns supreme. The public have absorbed from the empirical school the new ideas its adherents are promulgating, and are being misled. This will last but a little time, and then, as has been usual, it will all be forgotten, and some other idea will prevail.

It behooves us to stand fast. Our position is impregnable. By continuing steadfast in what we know to be right we shall gain more adherents, and thus the world will continue to be benefited by the genius of Hahnemann and the honesty of his followers.

G. H. C.

---

PROFESSOR KOCH'S DISCOVERY.—Hahnemannians possess, beside the law of cure, the only true idea regarding the *nature* of diseases. This knowledge can now be applied to Professor Koch's claims in respect to the cure of tuberculosis, and it will enable any follower of Hahnemann to pronounce a true verdict on the power of this so-called cure before all the allopathic evidence in its favor has been offered.

We do not hesitate to say that it will be found to be capable of doing only what any other one remedy can do; it has its curative sphere, and that sphere can only be known by making a proving of the substance used—no matter what it may be. After this is done we can show just what conditions of sickness it will cure. If this be done at once, and if Professor Koch and his adherents will accept it as indicating the power of his remedy—which we know they will not—we can assure them that their admirers will have less blame to throw upon them when they at last realize how they have been misled.

G. H. C.

---

DR. SWAN'S THEORY.—Dr. Swan should now take the floor and show that he has priority of claim; he can also show that he has a method of administering his remedy—thanks to the genius of Hahnemann—that will not jeopardize either life or health, as Koch's remedy is said to do.

Mingled with the amusement that is caused by Koch's widely published claims must be sorrow for those who are being buoyed up with the hope that their fatal disease can now be cured.

If the laity could only realize that the history of old-school medicine is but a series of crazes similar to the present one, there would be less harm from these unstable claims. But only a knowledge of homœopathic law can teach that empiricism is hurtful, and little that is good can come from such lawless procedures. G. H. C.

---

THE DEVELOPMENT OF NOSODE PRACTICE IN THE OLD SCHOOL.—The following is an editorial from a recent number of *The Medical Record*, of New York :

"Several recent studies upon the products of the growth or activity of pathogenic bacteria show that among these products are certain poisonous proteid bodies. These substances possess in some cases the same pathogenic property as in the original micro-organism. We have, therefore, as the result of microbic activity, not only ptomaines which are alkaloids, but a class of bodies which react like albumins.

"This latter class has been called pathogenic albumoses or 'tox-albumins.' Mr. E. H. Hankin, of Cambridge, has obtained such an albumose from anthrax culture, and Mr. Cartwright Wood has used it successfully as a protective vaccine. Brieger and Fraenkel have studied the diphtheria bacillus of Löffler, and have obtained a toxic albumin from it. They found that it produced no ptomaine.

"They obtained similar bodies from cultures of the specific microbes of typhoid, cholera, and tetanus, and of the staphylococcus pyogenes aureus, and in each case the bodies possessed definite pathogenic properties. The details given are not numerous, but it is of interest to note that the proteid isolated from cultures of staphylococcus appeared to give rise to the formation of pus differing from normal pus only in being completely devoid of micro-organisms.

"The practical importance of these discoveries lies in the fact that it may be possible, through the use of these new bodies, to obtain protective vaccines for various dieases."

That these bodies should possess "definite pathogenic properties" should excite no wonder in the mind of a follower of Hahnemann, but, if memory serves us, we have an impression that these men who now present "these bodies" have been heaping abuse upon Hahnemannians for using similar bodies for curing sicknesses in which only a true homœopathician can know how to scientifically use them—that is, by adhering to the law of similars, and properly proving them.

The following is of the same character :

"The *Journal de la Santé* relates that Dr. Babchinski, a Russian physician, having had his son affected with grave diphtheria, erysipelas of the face suddenly supervened, which was followed by a remarkable change in the state of the patient—the fever fell, the false membranes disappeared, and the patient was cured in a short time. Dr. Babchinski had observed in several other patients a similar improvement taking place after the disappearance of an attack of erysipelas, and in one of them the erysipelas had invaded the leg. These facts suggested to this physician the idea of inoculating a diphtheritic patient with blood taken from a patient affected with erysipelas. Erysipelas declared itself, things passed as in the preceding case, and the child which was inoculated was cured. Subsequently he practiced inoculations on other diphtheritic patients with cultures of microbes of erysipelas, cultivated on agar-agar, and constantly the manifestations of diphtheria disappeared. It may be added that, besides the inoculations, the patients had not been submitted to any other special medication whatever, and that in no case did erysipelas present any grave symptom. Dr. Babchinski concludes his note with the following remarks: 'If my observations and my experiments are confirmed, this treatment of diphtheria will be easy and certain, and this malady will no longer be dreaded.'"

Thus does scientific empiricism build up a therapy. We trust those who blindly follow in the footsteps of such teachings will now let us know upon what grounds they decry the proper use of any substance for the cure of disease.

Despite the opportunities offered these false scientists, in the works of Hahnemann and his followers, to become acquainted with the only real scientific method of learning what remedies for disease are capable of doing, they continue in their blindness. The darkness of the Middle Ages still obscures their vision.

Here is another specimen :

"At a recent meeting of the Academy of Medicine M. Nocard read a paper by M. Peyraud on *The Etiology and Treatment of Tetanus*. M. Peyraud, having inoculated a number of rabbits with an infusion which he made from hay, says he was able by this means to bring on an attack of tetanus in fifty per cent. of the animals inoculated. The animals thus inoculated succumbed in the proportion of five out of every six. M. Peyraud has a theory that a chemical substance capable of exciting symptoms analogous to those caused by the invasion of the system by a given micro-organism will prove by inoculation to be a vaccine against the ravages of the microbe. He has applied this theory to Strychnine, considered as the vaccine against tetanus. His method of proceeding was as follows: He injected hypodermically for a period of five or six days a dose of Strychnine, varying the dose according to the size of the animal and the appearance of the convulsions. The animals being thus prepared, he inoculated them with pus obtained from an animal previously dead of tetanus. Ten of such rabbits were inoculated, but, in addition to these ten already prepared, he inoculated, as a controlling experiment, four others not previously protected by Strychnine vaccination. The whole four non-vaccinated ones died and three of the ten vaccinated. The death of three of the prepared animals was attributed to a supplementary injection of Strychnine, which proved too strong. M. Nocard repeated these experiments by following a somewhat different method. He prepared a pure culture of tetanic bacilli from a lamb. Then he took ten rabbits and injected under the skin of each for five days in succession, ten drops of a solution of Sulphate of Strychnine of the strength of 1 in 1,000. He next inoculated the ten with his bacillary culture, controlling the experiment by at the same time inoculating ten untouched rabbits with the same culture. The result, however, was that they all died in from three to five days. He repeated the experiment with slight modifications, but the result was not less disastrous. The conclusion, therefore, was obvious."

What was "obvious"? Certainly the ignorance of the experimenter. "Ten drops of a solution of Strychnine of the strength of 1 in 1,000"! and "for five days in succession"! The next experiment he should make should be to try the same solution on himself in order to see what effect it would have on fools.

G. H. C.

---

The Future of Medicine.—The British Medical Association's meeting, held at Birmingham, in the last days of July, as usual produced some food for thought. From the words quoted below of Dr. Broadbent, of London, who gave an "Address on Therapeutics," we may learn what the future may give. Dr. Broadbent said: "I can lay claim to only one quality in accepting the honor, and that is, I have an immense interest in the subject as a branch of science, and not only as a professional means of gaining a living. I have a profound conviction that, in the therapeutic art, there must be fixed laws, if only these could be discovered, and that, sooner or later, the art of therapeutics will enter the scientific epoch, and be ranked with arts such as engineering or other arts which applied the exacter sciences to the benefit of mankind."

Great Scott!! This in the last decade of the nineteenth century, and from a leading London physician of the scientific (?) school!

---

This, from the same address, for the benefit of those who take the name of homœopathist, and still use palliatives: "It is bad practice to be continually using purgatives, stimulants, or narcotics. When the urine is turbid, it is not sufficient at once to prescribe an alkali, and it is short-sighted policy to continue giving bromides indefinitely in epilepsy. If this was done, the health of the patient might be deteriorated with no benefit to the fits. As much as possible, the practitioner must resist the desire for palliatives. One more protest I must raise—it is against the rage for new drugs which seems to have taken possession of the profession. This is absolutely fatal to accuracy ot observation and precision in treatment. * * * When drugs are recommended simply by an advertising chemist, it is humiliating to see such statements command general acceptance. May I take the dangerous liberty of indicating the points of my diagnosis of a weak medical man? They are indiscriminate administration of stimulants in fever, a ready resort to narcotics and sedatives, treatment directed to symptoms only, and a fondness for new drugs with high-sounding names." G. H. C.

